# Mechanisims of asthma and allergic disease – 1064. Interleukin 31 (IL 31) serum levels in atopic dermatitis patients

**DOI:** 10.1186/1939-4551-6-S1-P62

**Published:** 2013-04-23

**Authors:** Noor Suryani Mohd Ashari, Siti Nur Syuhada, M Mustaffa, Wan Zuraida Wah, AR Azriani, I Zulrushydi

**Affiliations:** 1 Universiti Sains Malaysia, Malaysia; 2 Hospital Raja Perempuan Zainab II, Malaysia

## Background

Atopic dermatitis is a common chronic skin disorder which is a subset of atopy. Atopy refers to a genetic predisposition to respond immunonologically to allergens which are generally harmless, causingover production of immunoglobulin E (IgE) antibodies. IgE synthesis is dependent on the activation of CD4+ Th2 subset and their secretion of cytokines including IL 4 and IL 13. This study was done to compare between IL 31 serum levels in normal controls and atopic dermatitis patients in Kelantan, Malaysia.

## Methods

This was a cross-sectional study of 34 samples of atopic dermatitispatients attending the skin clinic of Hospital Universiti Sains Malaysia (HUSM) and Hospital Raja Perempuan Zainab II (HRPZ II), Malaysia. 34 samples of normal controls were taken from healthy people (those who were free from allergic history) in HUSM. The subjects of healthy controls and atopic dermatitis patients were defined through history taking by physician. 5 ml of blood were collected from the normal control subjects and patients. Then, the blood was centrifuged and analyzed for IL 31 using ELISA kits (Human IL 31 Duoset, RnD System). Independent T-test was used to compare the level of serum IL 31 betweennormal control subjects and patients with atopic dermatitis.

## Results

The result showed that there was no significant difference in IL 31 serum levels between non allergic (174.89 ± 33.08, n=34, p=0.082) and atopic dermatitis patients (16499.46 ± 9243.56, n=34). However, there was a trend towards a higher IL31 serum levels in atopic dermatitis patients.

## Conclusions

The results of this study suggest that although the level of IL31 serum levels was higher, there was no significant difference in IL 31 serum levels between atopic dermatitis patients and non allergic control.

